# High prevalence of ventricular repolarization abnormalities in people carrying *TGFβR2* mutations

**DOI:** 10.1038/s41598-018-31298-5

**Published:** 2018-08-29

**Authors:** F. Extramiana, O. Milleron, S. Elbitar, A. Uccellini, M. Langeois, M. Spentchian, G. Delorme, F. Arnoult, I. Denjoy, C. Bouleti, V. Fressart, F. Iserin, P. Maison-Blanche, M. Abifadel, A. Leenhardt, C. Boileau, G. Jondeau

**Affiliations:** 1 CNMR Maladies Cardiaques Héréditaires Rares, Hôpital Bichat, 75018 Paris, France; 2 Université Paris Diderot, Sorbonne Paris Cité, F-75018 Paris, France; 3 AP-HP, Service de Cardiologie, Hôpital Bichat, F-75018 Paris, France; 4 CNMR Syndrome de Marfan et apparentés, Hôpital Bichat, 75018 Paris, France; 5 Laboratory of Biochemistry and Molecular Therapeutics, Faculty of Pharmacy, Saint-Joseph University, Beirut, Lebanon; 6 LVTS, INSERM U1148, Hôpital Bichat, F-75018 Paris, France; 7 AP-HP, Service d’Explorations Fonctionnelles, Hôpital Bichat, F-75018 Paris, France; 8 INSERM U956, Hôpital Pitié-Salpêtrière, F-75013 Paris, France; 9 AP-HP, Service de Biochimie Métabolique, Groupe Hospitalier Pitié-Salpêtrière, F-, 75013 Paris, France; 10 AP-HP, Hôpital Necker - Enfants Malades, F-75015 Paris, France; 11 AP-HP, Département de Génétique, Hôpital Bichat, F-75018 Paris, France

## Abstract

Mutations in the *TGFβR2* gene have been associated with a life threatening risk of aortic dissection but no arrhythmic death has been previously reported. Two young females carrying a *TGFβR2* mutation, initially diagnosed as Marfan syndrome or Loeys Dietz syndrome, presented sudden death with autopsy ruling out dissection. The ECGs of the 2 Sudden Cardiac Deaths revealed profound ventricular repolarization abnormalities with a sinusoidal T-U morphology associated with normal left ventricular ejection fraction. These data strongly suggest sudden cardiac arrhythmic deaths and prompted us to systematically study the repolarization pattern in the patients with *TGFβR2* mutations. ECG findings from 58 mutation carriers patients (*TGFβR2* group) were compared with those of 46 non-affected first degree relatives (control group). *TGFβR2* mutation was associated with ventricular repolarization abnormalities in 47% of patients (p < 0.001 vs. controls), including a 19.6 ms (95%CI 8.7; 30.5) QTc interval prolongation compared to the non-affected first degree relatives (p < 0.001), higher prevalence of abnormal U waves (16% vs. 2%), and sinusoidal T-U morphology (10% vs. 0%). *TGFβR2* mutations can be associated with abnormal ventricular repolarization pattern, longer QT interval than non-carrier relatives and an increased risk for sudden death.

## Introduction

Mutations in the *TGFβR2* gene have first been described by our group in patients diagnosed as having Marfan syndrome^1,2^. Since then, *TGFβR2* mutations have been associated with very variable phenotypes^3–6^. These mutations appear to be remarkable for their phenotypical variability which is only partly explained by genotype/phenotype correlation^7^.

The lethal risk associated with these mutations is primarily related to aortic dilatation/rupture^8^. To date, no arrhythmogenic risk has been associated with *TGFβR2* gene mutations. In contrast, Marfan syndrome related to mutations in the *FBN1* gene has been associated with a higher prevalence of ventricular arrhythmias as well as with arrhythmic deaths^9–12^.

We here report two sudden cardiac deaths (not related to arterial dissection) in 2 *TGFβR2* mutation positive young women seen in our center for Marfan Syndrome and related disorders.

The retrospective review of the two patient records revealed significant ventricular repolarization abnormalities and echocardiography bi-leaflet mitral valve prolapse. Electrocardiographic QT-T abnormalities (either as stand alone or in association with mitral valve prolapse) may reflect ventricular repolarization heterogeneities potentially serving as a substrate for malignant ventricular arrhythmias.

This prompted us to retrospectively evaluate ECG ventricular repolarization parameters and echocardiographic data in our *TGFβR2* cohort.

## Results

### Report of the 2 Sudden Cardiac Deaths

Two young women experienced sudden cardiac death with autopsy ruling out aortic dissection, brain aneurysm rupture or other macroscopic cause of death.

The first case occurred in a 17 year-old girl carrying the c.1182 C > G, p.Cys394Trp mutation (Supplemental Figure S1). At clinical examination, she presented hypertelorism, bifida uvula, arched palate, pectus carinatum, scoliosis, and spondylolisthesis. Echocardiography showed an aortic root diameter of 48 mm (174 cm, 47 kg); a symmetric bi-leaflet mitral valve prolapse with a trivial regurgitation was noted. Systolic ventricular function was normal and LVEDD 50 mm. She died suddenly a few days after prescription of mirtazapine, a tetracyclic anti-depressant drug known for possible torsade de pointes risk^13^.

**Figure 1:**
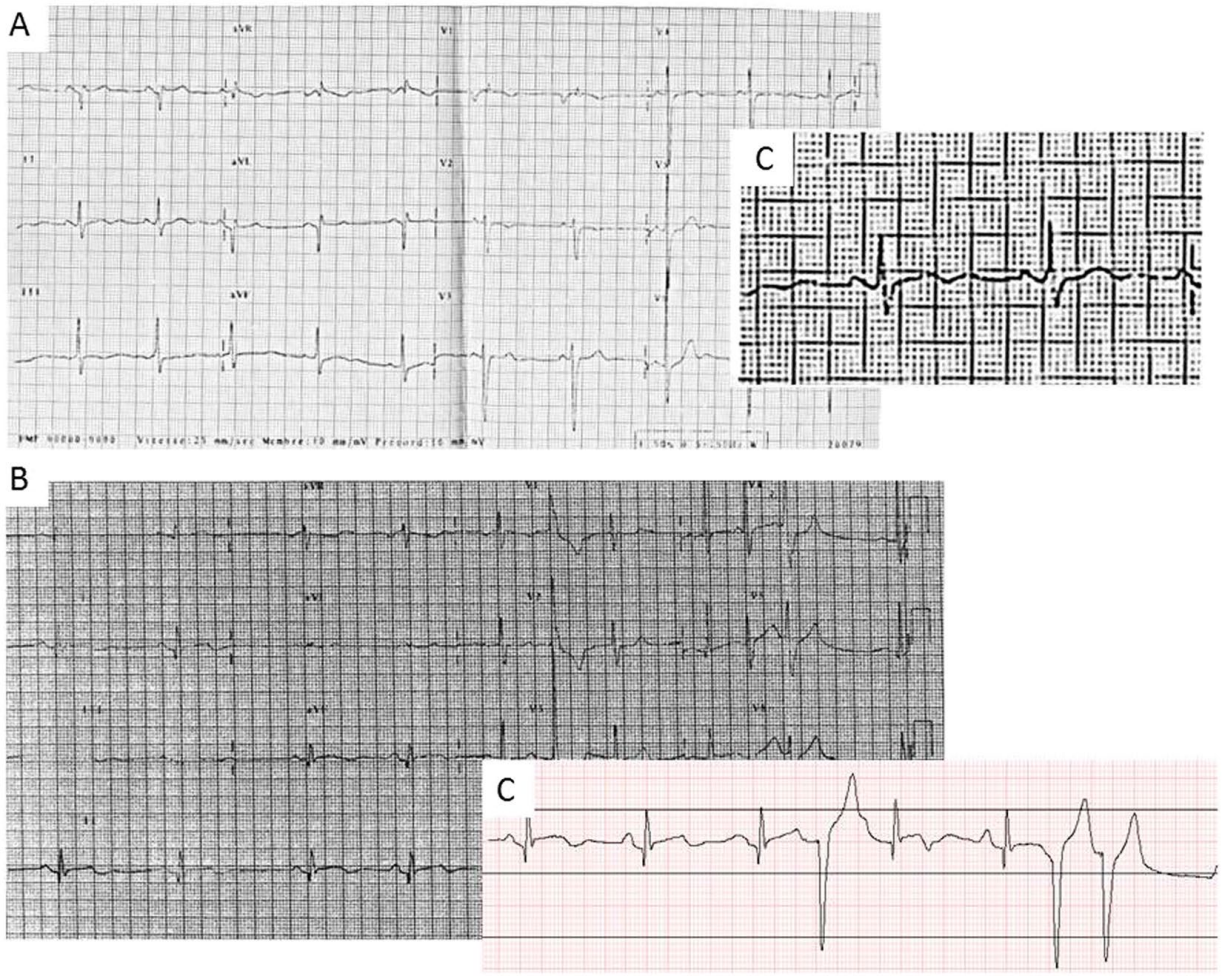
ECG of the 2 *TGFβR2* patients who presented sudden cardiac death. (**A**) 17 year-old woman carrying the c.1182 C > G -p.Cys394Trp mutation. Abnormal QRS axis and morphology and significant ventricular repolarization abnormalities with abnormal U waves but normal QTc duration (QTcB = 426 ms, QTcF = 427 ms). (**B**) Patient diagnosed as LQTS and Marfan syndrome with c.923 T > C -p.Leu308Pro mutation. ECG shows a sinus bradycardia and abnormal ventricular repolarization with sinusoidal T-U waves. QTcB = 440 ms, QTcF = 461 ms, abnormal U wave and ventricular premature beats. (**C**) Magnified lead II tracing in the 2 sudden death patients. Note the sinusoidal T-U waves morphology. ECG signal of Fig. 1B has been digitized for better quality tracing. Digitalization of Fig. 1A ECG tracing was not achievable.

ECG from the patient clinical file revealed significant ECG ventricular repolarization abnormalities with an abnormal U wave but normal QTc duration (QTcB = 426 ms, QTcF = 427 ms) (Fig. 1A) leading to a sinusoidal T-U waves morphology (Fig. 1C). Her mother and her sister carried the same mutation and presented aortic root dilatation as well as significant ventricular repolarization abnormalities (mother: QTcB = 485 ms, sister: marked U waves on inferior and lateral leads with QTcB = 410 ms).

The second Sudden Cardiac Death occurred in a 20 year-old young female in the context of intense stress (after a visit with a cardiac surgeon to prepare aortic surgery, and before a university exam). She had a history of resuscitated cardiac arrest at the age of 13. The diagnosis of LQTS had been then retained, and at that time (1993) she was prescribed beta-blocker therapy only (i.e. no ICD). Because skeletal features (arched palate, arachnodactyly, pectus carinatum, hyperlaxity, protrusia acetabula) led to the suspicion of Marfan Syndrome, she had been referred to our center and Marfan syndrome was diagnosed on a clinical basis. ECG showed profound ventricular repolarization abnormalities with sinusoidal T-U waves morphology (QT = 507 ms at RR = 1326 ms, QTcB = 440 ms, QTcF = 461 ms) and monomorphic ventricular premature beats (Fig. 1B and C). The echocardiography showed an aortic root diameter of 48 mm (19 years, 180 cm, 55 kg) with minimal aortic regurgitation, a bi-leaflet mitral valve prolapse with mild regurgitation, and a left ventricular dilatation (LVEDD 66 mm) with preserved ejection fraction. She was treated with mesalazine for ulcerative colitis. She presented a *de-novo* mutation in the *TGFBR2* gene: c.923 T > C, p.Leu308Pro (Supplemental Figure S1) and her first degree relatives had normal ECG.

Whole exome sequencing was performed in these 2 patients. No significant variant was found in any of the genes associated with LQTS in the first patient. A rare variant (c.1088 A > C, p.Glu363Ala) was found in the *SNTA1* gene associated with type 12 LQTS in the second patient. This variant has been reported at a frequency of 0.04% in ExAC. The mother was also a carrier for this variant whereas her ECG is normal and no indication for arrhythmia was reported. Therefore it was concluded that no mutation other than that of the *TGFβR2* gene could explain the LQTS responsible for the sudden death of these 2 patients.

### Evaluation of *TGFβR2* mutation positive patients and their relatives

ECG tracings suitable for measurements were obtained in 58 patients (from 22 families) with a *TGFβR2* mutation (Supplemental data Table 1). Among these patients, 32% had aortic tortuosity, 61% cervical artery tortuosity, 56% cleft palate and 24% bifid uvula, 7% hypertelorism, 62% wide scars. Sixteen percent had a “systemic score” as proposed in the last Ghent nosology (14) greater or equal to 7 (mean score 3.9 ± 2.9). Aortic root diameter was 40 ± 8 mm (z-score 4.6 ± 3.5).

Clinical data, echographic data and ECG were available in 46 non-carrier first degree relatives. The 2 groups were comparable with the exception of a higher proportion of beta blocker treatment and larger aortic diameters in *TGFβR2* mutation carriers (Table 1).

**Table 1:** Clinical, echographic and ECG data *TGFβR2* group versus control group.

		Mutation positive (n = 58 patients)	Non carriers (n = 46 relatives)	p
Clinical data	Age	29.1 ± 16.7	32.6 ± 15.8	0.27
	Sex (F/M)	30/28	24/22	0.99
	Height (cm)	171 ± 14	167 ± 18	0.15
	Weight (kg)	62 ± 19	66 ± 20	0.11
	Body surface (m²)	1.71 ± 0.30	1.72 ± 0.34	0.89
	Body Mass Index	20.7 ± 5.2	23.2 ± 6.1	0.03
	Beta blockade	58%	2%	<0.001
Echographic data	LVEDD (mm)	49.4 ± 7.5	49.1 ± 4.9	0.83
	Indexed LVEDD(mm/m²)	29.8 ± 6.2	28.8 ± 5.6	0.38
	LVESD (mm)	30.9 ± 6.5	30.3 ± 3.4	0.71
	Indexed LVESD (mm/m²)	18.6 ± 4.4	17.8 ± 3.8	0.36
	LVEF (%)	66.2 ± 10.4	68.0 ± 5.5	0.29
	Aortic Root diameter (mm)	40.0 ± 7.7	30.5 ± 4.4	<0.001
ECG measurements	RR (ms)	968 ± 199	908 ± 140	0.09
	QT (ms)	402 ± 41	372 ± 31	<0.001
	QTc Bazett (ms)	411 ± 31	392 ± 23	<0.001
	QTc Fridericia (ms)	408 ± 29	385 ± 23	<0.001
Ventricular repolarization qualitative evaluation	Early repolarization Of which Marked	22% 16%	13% 2%	0.31 <0.05
	Abnormal U wave	16%	2%	<0.05
	Prolonged QTc (>450 ms)	14%	0%	<0.05
	Abnormal ST-T morphology Sinusoidal T-U morphology	24% 10%	7% 0%	<0.05 <0.05
	Significant abnormality	47%	11%	<0.001

#### ECG findings

**Figure 2:**
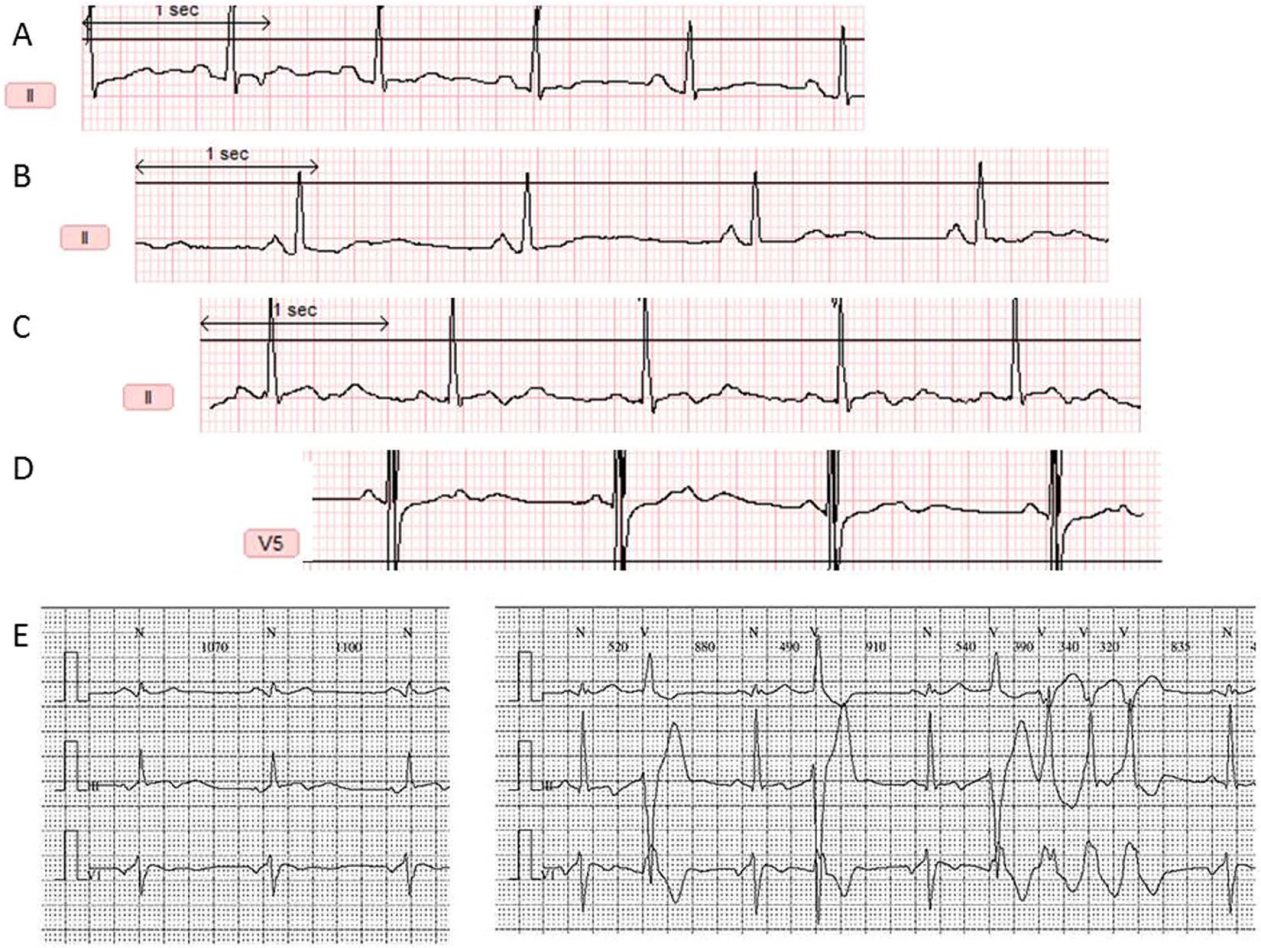
Sinusoidal T-U waves morphology in 4 additional patients with *TGFβR2* mutations. (**A**) 32 year-old women, p.Arg537Cys mutation, QTcB = 466 ms, LVEF 73%, LVEDD 41 mm, normal mitral valve. (**B**) 42 year-old women, p.Q511X mutation, QTcB = 435 ms, LVEF 70%, LVEDD 50 mm, mitral valve prolapse. (**C**) 15 year-old boy, p.Asp524Asn mutation, QTcB = 381 ms, normal LVEF, LVEDD 58 mm, mitral valve prolapse. (**D**) 27 year-old woman, p.Asn384Asp mutation, QTcB = 425 ms, normal LVEF, LVEDD 53 mm, normal mitral valve. (**E**) Holter ECG of a 15 year-old boy carrying the p.Asp524Asn mutation. Upper part: abnormal T-U wave morphology with prominent U wave. Lower part: right bundle branch block morphology ventricular run. ECG signal of Fig. 2A–D have been digitized for better quality tracing.

Conduction abnormalities were observed in 5/58 mutation carriers (2 patients with complete and 3 with incomplete right bundle branch block) and in 2/46 relatives (incomplete right bundle branch block) (p = 0.45). QT and QTc interval durations were significantly longer in *TGFβR2* mutation positive patients than in their non-affected relatives (Table 1, Supplemental Figure S2). A QTc >450 ms was observed in 8 patients (13.8%) of the *TGFβR2* group (4 females) versus none in the control group (p < 0.05). Of note, 4 out of the 8 ECG with prolonged QTc interval were recorded after aortic surgery had been performed years ago. Significant ventricular repolarization abnormalities other than prolonged QTc were also more frequent in *TGFβR2* mutation carriers than in their non-affected first degree relatives (Table 1). The sinusoidal T-U wave morphology described above in the 2 sudden cardiac deaths was also observed in 4 additional patients with *TGFβR2* mutations (Fig. 2). One of these 4 patients (Fig. 2 – panel C) had frequent and repetitive ventricular premature beats on Holter recording (Fig. 2 – panel E). Among these 4 patients with sinusoidal T-U wave morphology, 2 had a mitral valve prolapse (Fig. 2, panel B and C) but the mitral valve was considered as normal in the 2 other patients (Fig. 2A and D).

Fourteen out of the 58 patients (24%) had a mitral valve prolapse (MVP). The presence of MVP was not associated with prolonged QT/QTc duration. However, the sinusoidal T-U wave morphology was more frequently observed in the presence of mitral valve prolapse (Supplemental Table 2) and MVP was present in the 2 patients with presumed arrhythmogenic sudden cardiac death. However, among the 9 patients with abnormal U waves, 5 (56%) had a MVP but the remaining 4 had a normal mitral valve.

Patients with significant ventricular repolarization abnormalities had larger aortic root diameter. Other parameters were not significantly different between the 2 subgroups (Table 2).

**Table 2:** Clinical and echocardiographic data in *TGFβR2* mutation carriers with or without significant ventricular repolarization abnormalities.

	*TGFBR2* Mutation positive patients	Significant Repolarization abnormality n = 27	Normal Repolarization Pattern n = 31	p
Clinical data	Age	27.2 ± 15.0	30.6 ± 18.1	0.44
	Sex (% females)	59%	45%	0.31
	Height (cm)	172 ± 10	171 ± 17	0.25
	Weight (kg)	60 ± 20	64 ± 19	0.42
	Body surface (m²)	1.69 ± 0.26	1.74 ± 0.34	0.51
	Body Mass Index	20.1 ± 6.0	21.2 ± 4.5	0.44
	Beta blockade	58%	58%	>0.99
Echographic data	EDD (mm)	50.5 ± 8.0	48.5 ± 7.0	0.32
	Indexed EDD	30.7 ± 5.6	29.0 ± 6.7	0.09
	ESD (mm)	32.2 ± 6.8	29.8 ± 6.0	0.17
	Indexed ESD	19.5 ± 3.9	17.8 ± 4.6	0.14
	LVEF (%)	65.2 ± 9.0	67.0 ± 11.5	0.54
	Valsalva sinus diameter	43.3 ± 7.2	37.2 ± 7.1	<0.01
	Mitral valve Prolapse	33%	17%	0.27

The determinants of QTc duration in our study population are described in supplemental data.

## Discussion

We report for the first time 2 Sudden Cardiac Deaths most probably due to ventricular arrhythmias occurring in 2 young females carrying a different *TGFβR2* mutation.

Retrospective analysis of their files revealed profound ventricular repolarization abnormalities with a sinusoidal T-U morphology on the ECG associated with normal left ventricular ejection fraction but with mitral valve prolapse.

Subsequent evaluation of our *TGFβR2* mutated patients and their relatives showed that presence of a *TGFβR2* mutation is associated with more prevalent ventricular repolarization abnormalities including prolonged QTc interval, abnormal U waves and in up to 10% with a sinusoidal T-U morphology.

Taken together, these data strongly suggest a clinical link between *TGFβR2* mutations, impaired ventricular repolarization and a potential risk for arrhythmic sudden death.

Although ECG was not recorded at the time of the Sudden Cardiac Death of our 2 young patients, an arrhythmic origin of the catastrophic events is highly likely.

Indeed, autopsy was performed in both cases and could rule out aortic dissection, brain aneurysm rupture or other macroscopic cause of death. Lethal ventricular arrhythmias can take place during acute myocardial ischemia (i.e. coronary spasm), complicate structural heart diseases or be the consequence of primary “electrical” diseases^14^. Our 2 patients could unfortunately not undergo a post Cardiac Arrest work-up to determine its cause. However, their pre-event histories share remarkably common features (*TGFβR2* mutations, bi-leaflet mitral valve prolapse and profound ventricular repolarization abnormalities with a sinusoidal T-U morphology on the ECG). The origin of this ventricular repolarization impairment will be discussed later, but certainly suggests the presence of a ventricular arrhythmic substrate hence further supporting the hypothesis of a Sudden Arrhythmic Death in our 2 patients.

Our ECG findings in the 2 SCD patients prompted us to evaluate ventricular repolarization in our *TGFβR2* cohort. When compared to mutation negative relatives, *TGFβR2* mutated patients were characterized by a high proportion of patients (almost 50%) showing significant ventricular repolarization abnormalities on their ECG. QTcB interval was prolonged by 19.6 ms (95%CI 8.7; 30.5) and 1/8 had a QTc prolongation >450 ms). This QTc prolongation cannot be explained by a QT correction bias or by confounding factors such as sex, age and beta-blocker treatment which have been shown to influence ventricular repolarization^15,16^. In the setting of regulatory evaluation of drug effect on ventricular repolarization, an upper one-sided 95% Confidence Interval of QTc prolongation effect >10 ms would be considered as significant^17^. Hence, the mean 20-ms QTc prolongation observed in patients with *TGFβR2* mutations cannot be considered as trivial. The mean QTc observed is not per se sufficient to be associated with a high risk of sudden arrhythmic death but may reflect a decreased repolarization reserve. According to guidelines and despite uncertainties in repolarization waves naming, we have carefully excluded any potential U-wave for QT interval measurement. A less strict (artificial?) evaluation of ventricular repolarization duration would have found higher proportion of QTc >450 ms and even QTc >500 ms in some patients (see Figs 1 and 2 for examples).

In addition, sinusoidal T-U waves were observed in 4 additional patients without history of SCD.

Our data clearly demonstrate the high prevalence of ventricular repolarization abnormalities in *TGFβR2* mutated patients. However, the cause of repolarization impairment cannot be ascertained from our data.

By definition, secondary ventricular repolarization abnormalities are the consequence of structural heart diseases. *TGFβR2* mutation carriers can present a cardiac involvement associated with repolarization abnormalities^5^. However, we found no relationship between the QT duration and the left ventricular dilatation or left ventricular ejection fraction. In addition, although LV was dilated in one of the SCD patient, it was not in the other and the 6 patients with the sinusoidal T-U morphology had a normal LV ejection fraction.

The relation between ventricular repolarization impairment and the presence of a mitral valve prolapse is more difficult to decipher. ECG abnormalities described in the malignant form of mitral valve prolapse^18,19^ are not very different from those we describe in our *TGFβR2* mutated patients. *TGFβR2* mutations are known to be associated with the presence of bi-leaflet MVP^5^ and MVP was present in 24% of our patients (i.e. 10-fold the estimated prevalence in the general population). In addition, MVP was present in both patients who died suddenly and in 2 out of the 4 other *TGFβR2* patients showing sinusoidal T-U morphology suggesting that *TGFβR2* gene mutations can favor sudden death through occurrence of MVP and its associated arrhythmogenic risks. On the other hand, among the 9 patients with abnormal U waves, 5 had a MVP but the remaining 4 had a normal mitral valve. The proportion of MVP was not significantly different between patients with or without ventricular repolarization abnormalities and QTc duration was not different according to the presence or absence of MVP. Finally, a higher proportion of MVP in *FBN1* patients was associated with a lesser degree of QTc prolongation than in *TGFβR2* mutation carriers (data not shown).

The results of our study may suggest that *TGFβR2* mutations can be associated with increased risk of sudden death though 2 additive paths: 1) the greater occurrence of MVP (more frequent in *TGFβR2*) may convey a higher risk of sudden death as in the non *TGFβR2* population 2) in addition, *TGFβR2* per se may independently be responsible for repolarization abnormalities and increase the risk of sudden deaths. This would be supported by the presence of repolarization abnormalities in patients without MVP.

Although it is difficult to exclude a relationship between structural and ECG abnormalities, primary ventricular repolarization abnormalities associated with *TGFβR2* mutations cannot be ruled out, and the QT duration in the whole population suggests a direct effect of *TGFβR2* mutation on repolarization (Supplemental Figure S2).

Recent data support the concept of an interaction between the TGFβ signaling pathway and the ventricular repolarization process^20–22^. Chu *et al*. demonstrated that TGF-β1 inhibited the expression of hERG and down-regulated Kir2.1 protein level, thus reducing I_Kr_ and I_K1_ repolarizing currents^21^. The loss of function of I_Kr_ and I_K1_ are the mechanism underlying type 2 and 7 congenital LQTS, respectively. Hence, these finding are hypothesis generating, including, but not limited to 1) a direct influence of TGFβ signaling pathway on ventricular repolarization, 2) a genetic modulation of the LQTS phenotype^23^, but also 3) an uncoupling effect of an abnormal fibrosis leading to pro-arrhythmic increase in ventricular repolarization dispersion^24^.

A direct effect of a *TGFβR2* mutation on ventricular repolarization would be further supported by the presence of a pathogenic *TGFβR2* mutation in LQTS patients. However, the search for *TGFβR2* mutations in *KCNQ1*, *KCNH2* and *SCN5A* genotype negative LQTS patients, only retrieved a variant of unknown significance in *TGFβR2* (Supplemental data).

Whatever the mechanisms involved in the repolarization abnormalities observed in our *TGFβR2* patients, our results underscore the potential life-threatening effect of drugs that prolong QT interval in these patients. Such a prescription preceded one of the 2 sudden deaths in a patient with repolarization abnormalities but normal baseline QTc. This peculiar risk with QT-prolonging drugs and stress-related events are shared features with the congenital LQTS^13,25,26^. In addition, by analogy with the congenital LQTS, a beta blocker therapy should be considered as the first line therapy when baseline ventricular repolarization is abnormal and/or when a therapy is considered to prevent aortic dilation^27^.

The retrospective nature of our observational study is associated with intrinsic limitations. We have tried to minimize these limitations by using blind readings and measurements of the ECGs.

The main limitation of our study is the lack of direct link between ECG ventricular repolarization abnormalities and arrhythmic events. Holter ECG recordings were available in 21/58 of our patients. Three patients (14%) (our first sudden cardiac death case, her mother and a 15 year-old boy carrying the c.1570 G > A mutation (Fig. 2E)) had significant ventricular arrhythmias (more than 500 VPBs and ventricular triplets or non-sustained runs). Functional studies and prospective follow-up are warranted to ascertain the pathophysiological link between TGF-β signaling, ventricular repolarization abnormalities and arrhythmic events.

It is premature to conclude that this aspect is associated with a risk of sudden death. Still, it underlines that such ventricular repolarization morphology abnormalities can be worrisome despite a normal QTc interval duration.

In conclusion, malfunction of type 2 *TGFβ* receptor is associated with a life-threatening risk of aortic dissection, but also life-threatening risk of ventricular arrhythmias that might be precipitated by QT-prolonging drugs. Prospective follow-up studies are on-going to demonstrate that the *TGFβR2* gene mutations are causative of the increased arrhythmia prevalence and risk of SD in this population. In the meantime, it would be important to promote further experimental evaluation of the link between the TGFβ signaling pathway and repolarization of ventricular cardiomyocytes.

Pending, it seems sensible that patients carrying a *TGFβR2* gene mutation should be informed about the risk of using QT prolonging drugs^13^, and that these drugs should be avoided when possible, or if mandatory, prescribed under strict ECG and medical supervision.

## Patients and Methods

### Subjects and patients

*TGFβR2* mutation carriers were evaluated at the French reference center for Marfan Syndrome and related disorders^12^. All patients underwent comprehensive clinical evaluation, with ECG, echocardiography, and screening for the Ghent criteria for Marfan syndrome and for features associated with LDS once it was described^4,28,29^.

All the patients, including the 2 SCD cases, with ECG quality suitable for interval measurements were included in the study. In order to minimize background genetic variability not related to *TGFβR2*, mutation negative relatives were used as controls.

Genetic analyses are described in the supplemental data. In accordance with the French law, patients or parents have signed a written informed consent for genetic analyses and granted permission of usage of their clinical data medical research analysis. Medical data were de-identified and handled according to French Data Protection Authority (CNIL) guidelines.

### ECG analysis

12-lead paper ECGs were scanned to obtain digital ECG images. All ECG reading and measurements were performed (blindly to the genetic status) by AU and FE after standardization of the procedures. Measurements were performed on 3 consecutive QRS-T complexes using on screen calipers (Rigel software, AMPS LLC, NY, NY). The end of the T-wave was determined using the tangent method or in case of U wave at the nadir between the T and U waves. Mean RR and mean QT intervals were used to calculate the QTcB and QTcF interval using Bazett’s and Fridericia’s formulae respectively^30^.

Early repolarization pattern was defined by the presence of J-point elevation ≥0.1 mV (“marked” when ≥0.2 mV) in ≥2 contiguous inferior and/or lateral leads^31^.

The U wave was considered as abnormal based on amplitude and/or percentage of the preceding T-wave amplitude as recommended by the AHA/ACCF/HRS Scientific Statement^30^.

ST segment and T wave abnormalities (ST segment shift, inverted T-wave, flat or huge T-wave) were collected.

Marked early repolarization pattern, abnormal U wave, abnormal ST segment and/or T wave and QTc >450 ms were considered as “significant ventricular repolarization abnormalities”^30^.

### Statistical analysis

Data are presented as mean ± SD. The normality of the distribution was evaluated using a Kolmogorov-Smirnov test. Differences between groups were compared using Student’s t test when data were normally distributed and using a Mann-Whitney test if not. The relationships between quantitative parameters were analyzed by Pearson coefficient. Qualitative parameters were compared using a Fisher’s exact test. A p value < 0.05 was considered significant.

## Electronic supplementary material

Supplementary data

## Data Availability

All the data used to obtain the results presented in this manuscript are available.
