# Supplementary material for: High prevalence of ventricular repolarization abnormalities in people carrying TGFβR2 mutations
Source: Sci Rep. 2018 Aug 29;8:13019. doi: 10.1038/s41598-018-31298-5 (PMC6115378; doi:10.1038/s41598-018-31298-5)
Supplement: Supplementary file 1 — Supplementary data [file 41598_2018_31298_MOESM1_ESM.pdf]

High prevalence of ventricular repolarization abnormalities in people carrying *TGFβR2* mutations

Extramiana F, M.D., Milleron O, M.D., Elbitar S, PharmD .D., Uccellini A, M.D., Langeois M, B.Sci., Spentchian M, B.Sci., Delorme G, M.D., Arnoult F, M.D., Denjoy I, M.D., Bouleti C, M.D., Fressart V, M.D., Iserin F, M.D., Maison-Blanche P, M.D., Abifadel M, Ph.D., Leenhardt A, M.D., Boileau C, Ph.D., Jondeau G, M.D..

Supplemental data

Mutation Analysis

Blood samples were obtained after written informed consent had been signed by patients or parents. All 7 exons of the *TGFβR2* gene, as well as close flanking intronic sequences, were amplified by PCR. Unidirectional sequencing for quick screening of the gene followed by bidirectional sequencing of altered exons was carried out as previously reported (Stheneur C, Collod-Béroud G, Faivre L, et al. Identification of 23 TGFBR2 and 6 TGFBR1 gene mutations and genotype-phenotype investigations in 457 patients with Marfan syndrome type I and II, Loeys-Dietz syndrome and related disorders. Hum Mutat 2008; 29: E284–295).

The population frequency of the variants found by sequencing was estimated using Exome Variant Server (evs.gs.washington.edu/EVS/), dbSNP (ncbi.nlm.nih.gov/SNP/), ExAc browser (exac.broadinstitute.org/) and the locus specific database UMD-*TGFβR2* (umd.be/*TGFBR2*). The causal effect of each new molecular event was estimated using Polyphen

(genetics.bwh.harvard.edu/pph), SIFT (sift.jcvi.org), MutationTaster (mutationtaster.org) and, ALAMUT.

Screening for *TGFβR2* mutations in the LQTS population

Among our LQTS patient database (n ≈ 1 000), we selected families with a clinical LQTS diagnosis suspicion but negative mutation screening in *KCNQ1*, *KCNH2* and *SCN5A*, the 3 main genes responsible for LQTS type 1, 2 and 3 respectively (Priori SG, Wilde AA, Horie M,et al. HRS/EHRA/APHRS expert consensus statement on the diagnosis and management of patients with inherited primary arrhythmia syndromes: document endorsed by HRS, EHRA, and APHRS in May 2013 and by ACCF, AHA, PACES, and AEPC in June 2013. Heart Rhythm 2013; 10: 1932–1963). One subject per family was screened for the presence of a *TGFβR2* mutation.

Sixty patients with suspected LQTS were screened for TGFβR2 gene mutations (full data available in 57). Mean age was 46.7±19.6 years, 79% were females, 79% were probands and 37% had experienced a resuscitated sudden cardiac arrest. Mean QTcB was 501±64 ms, and the LQTS diagnostic Schwartz criteria score was 4.1±1.9.

A new TGFβR2 gene variation was identified in a 60 year-old woman diagnosed as LQTS in 2001 after a first syncope episode (QTcB=480ms, Schwartz score =4). The syncope had occurred while on cetirizine a selective inhibitor of peripheral histamine (H1) receptors, which has been associated with a QT prolongation (Poluzzi E, Raschi E, Godman B, Koci A, Moretti U, Kalaba M, Wettermark B, Sturkenboom M, De Ponti F. Pro-arrhythmic potential of oral antihistamines (H1): combining adverse event reports with drug utilization data across Europe. PLoS One 2015; 10: e0119551). She was then advised to avoid QT-prolonging drugs and to take beta-blocker therapy.

This variation is a substitution p.Glu485Lys (c.1453G>A) in exon 6 of TGFβR2, located in the highly conserved serine/threonine kinase domain of the receptor (Figure 1) and has not been reported in Exome Variant Server, dbSNP, and ExAc databases. In silico, this variant is predicted to be damaging using SIFT tools (score of 0.001), to be probably damaging using Polyphen (score of 0.993) and, disease causing using Mutation taster (score of 56). Unfortunately, no segregation analysis could be performed in the family.

A complete new clinical evaluation of the patient was performed; No feature of LDS could be found except a wide uvula. The aortic root diameter measured on echocardiography was normal (32 mm), as was the mitral valve. A CT scan of all the vessels did reveal no aneurysm or other abnormality.

ECG analysis process

Paper ECGs were retrieved from medical files and scanned in a TIFF image format (black and white, 300 dpi).

The ECG image was then opened using the Rigel software. We used a 4-fold magnification of ECG tracing, calibrated the x axis (time) and then measured intervals as described in the method section.

Determinant of QTc duration

The Fridericia's correction formula was less rate dependent than the Bazett's in both *TGFβR2* mutations positive patients (QTcF=0.009*RR+399, R^2=0.003, p=0.66 and QTcB =–0.059*RR+469, R^2=0.15, p<0.01) and the control group and was used for correlation studies. In *TGFβR2* mutation carriers, QTcF was slightly positively correlated with age (R^2=0.08, p<0.05)

and weight (R^2=0.08, $p<0.05$) but not with height, body surface or body mass index. QTcF was also correlated with aortic root diameter but not with z-score or any other echocardiographic parameter in this population.

Supplemental Table 1. Mutation, aortic history and death in the 58 *TGFBR2* gene mutation carriers

Patient	Family	Sex	Age at echo	*TGFBR2* mutation		Cause of death (age)	Aortic surgery (age)	Aortic diameter (mm)
				Nucleotide position	Protein position			
1	1	female	63	c.1524G>A	p.Gln508Gln		AAD (58)	
2	1	female	41	c.1524G>A	p.Gln508Gln	AAD (50)	DAD (41)	
3	1	female	15	c.1524G>A	p.Gln508Gln			31
4	1	male	16	c.1524G>A	p.Gln508Gln			37
5	1	female	15	c.1524G>A	p.Gln508Gln			33
6	1	male	30	c.1524G>A	p.Gln508Gln			47
7	1	female	30	c.1524G>A	p.Gln508Gln			38
8	1	male	25	c.1524G>A	p.Gln508Gln			41
9	1	male	61	c.1524G>A	p.Gln508Gln			47
10	1	female	63	c.1524G>A	p.Gln508Gln			33
11	1	female	25	c.1524G>A	p.Gln508Gln			36
12	1	female	42	c.1524G>A	p.Gln508Gln			43
13	1	female	8	c.1524G>A	p.Gln508Gln			32
14	1	female	36	c.1524G>A	p.Gln508Gln			29
15	1	female	42	c.1524G>A	p.Gln508Gln			33
16	1	male	13	c.1524G>A	p.Gln508Gln			36
17	1	female	67	c.1524G>A	p.Gln508Gln		AAD & DAD (63)	
18	1	male	12	c.1524G>A	p.Gln508Gln			36
19	1	female	7	c.1524G>A	p.Gln508Gln			28
20	1	male	8	c.1524G>A	p.Gln508Gln			28
21	1	female	16	c.1524G>A	p.Gln508Gln			33
22	1	female	32	c.1524G>A	p.Gln508Gln			44
23	1	female	27	c.1524G>A	p.Gln508Gln			37
24	57	female	19	c.923T>C	p.Leu308Pro	SCD (20)		48
25	219	male	21	c.1273A>G	p.Met425Val		AAD & DAD (31)	49
26	261	male	40	c.1489C>T	p.Arg497X		A dilation (29)	49

27	261	female	59	c.1489C>T	p.Arg497X			32
28	284	male	46	c.1609C>T	p.Arg537Cys		AAD (36)	
29	284	female	22	c.1609C>T	p.Arg537Cys			34
30	284	female	17	c.1609C>T	p.Arg537Cys			33
31	382	female	11	c.1346C>T	p.Ser449Phe		A dilation (12)	50
32	587	male	28	c.1609C>T	p.Arg537Cys		A dilation (31)	38
33	587	male	45	c.1609C>T	p.Arg537Cys			51
34	587	female	32	c.1609C>T	p.Arg537Cys		AAD & DAD (34)	59
35	587	male	17	c.1609C>T	p.Arg537Cys			30
36	587	male	4	c.1609C>T	p.Arg537Cys			43
37	752	female	42	c.1531C>T	p.Gln511X			48
38	816	male	14	c.1582C>T	p.Arg528Cys			49
39	1066	female	14	c.1182C>G	p.Cys394Trp			41
40	1066	female	18	c.1182C>G	p.Cys394Trp	SCD (17)		48
41	1066	female	45	c.1182C>G	p.Cys394Trp		A dilation (30)	47
42	1135	male	39	c.1336G>A	p.Asp446Asn		A dilation (32)	
43	1149	male	15	c.1570G>A	p.Asp524Asn			46
44	1228	male	43	c.1379G>A	p.Arg460His		A dilation (37)	42
45	1228	female	4	c.1379G>A	p.Arg460His			23
46	1454	male	44	c.1379G>A	p.Arg460His		DAD & A dilation (33 and 38)	
47	1733	male	33	c.964T>G	p.Trp322Gly			39
48	1733	male	52	c.964T>G	p.Trp322Gly		DAD & A dilation (49 and 50)	45
49	1733	male	19	c.964T>G	p.Trp322Gly			38
50	1803	female	45	c.1483C>T	p.Arg495X	Cancer (49)		45
51	2161	female	26	c.1658C>T	p.Ser553Leu			47
52	2406	male	24	c.1379G>A	p.Arg460His			40
53	2530	female	27	c.1150A>G	p.Asn384Asp		AAD (22)	
54	2530	male	19	c.1150A>G	p.Asn384Asp		A dilation (15)	44
55	2530	female	10	c.1150A>G	p.Asn384Asp			43
56	2813	male	54	c.1032G>C	p.Trp344Cys			49
57	3462	male	36	c.1609C>T	p.Arg537Cys		AAD & DAD (28 and 36)	43
58	3462	male	7	c.1609C>T	p.Arg537Cys			26

AAD: ascending aortic dissection

DAD: descending aortic dissection

A dilation: aortic dilation

SCD: sudden cardiac death

Supplemental Table 2. ECG data according to mitral valve morphology in *TGFβR2* mutation positive patients

	Normal Mitral Valve n=45	Mitral Valve Prolapse n=13	p
RR (ms)	950±190	1044±233	0.15
QT (ms)	398±39	416±49	0.19
QTcB (ms)	412±29	410±39	0.86
QTcF (ms)	407±27	412±38	0.62
Abnormal U wave	11%	31%	0.08
Prolonged QTc (>450 ms)	11%	23%	0.34
Sinusoidal T-U morphology	4%	31%	<0.05

Supplemental Figure S1

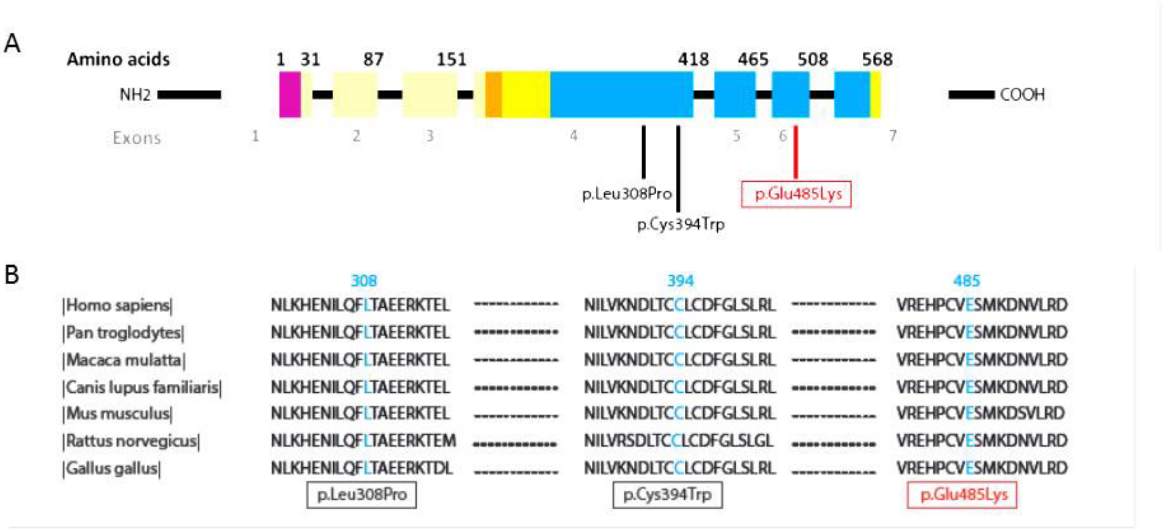

Supplemental Figure S1

Structure of the *TGFβR2* and reported mutations

A- *TGFβR2* consists of 7 exons. The different domains of the protein are shown in colors. The mutations in black are those found in the 2 deceased *TGFBR2* mutation carriers. The mutation p.Glu485Lys in red is the one found in a 60 year-old woman with suspected LQTS and negative screening in *KCNQ1*, *KCNH2* and *SCN5A* genes. All mutations are located in the serine/threonine kinase domain of the receptor as those most often found.

B- All of the 3 substitutions found in our patients (c.923T>C- p.Leu308Pro, c.1182C>G- p.Cys394Try, and c.1453G>A-p.Glu485Lys) affect an amino acid evolutionarily conserved among different species. Multiple sequence alignments were performed with CLUSTAL Omega

Supplemental Figure S2

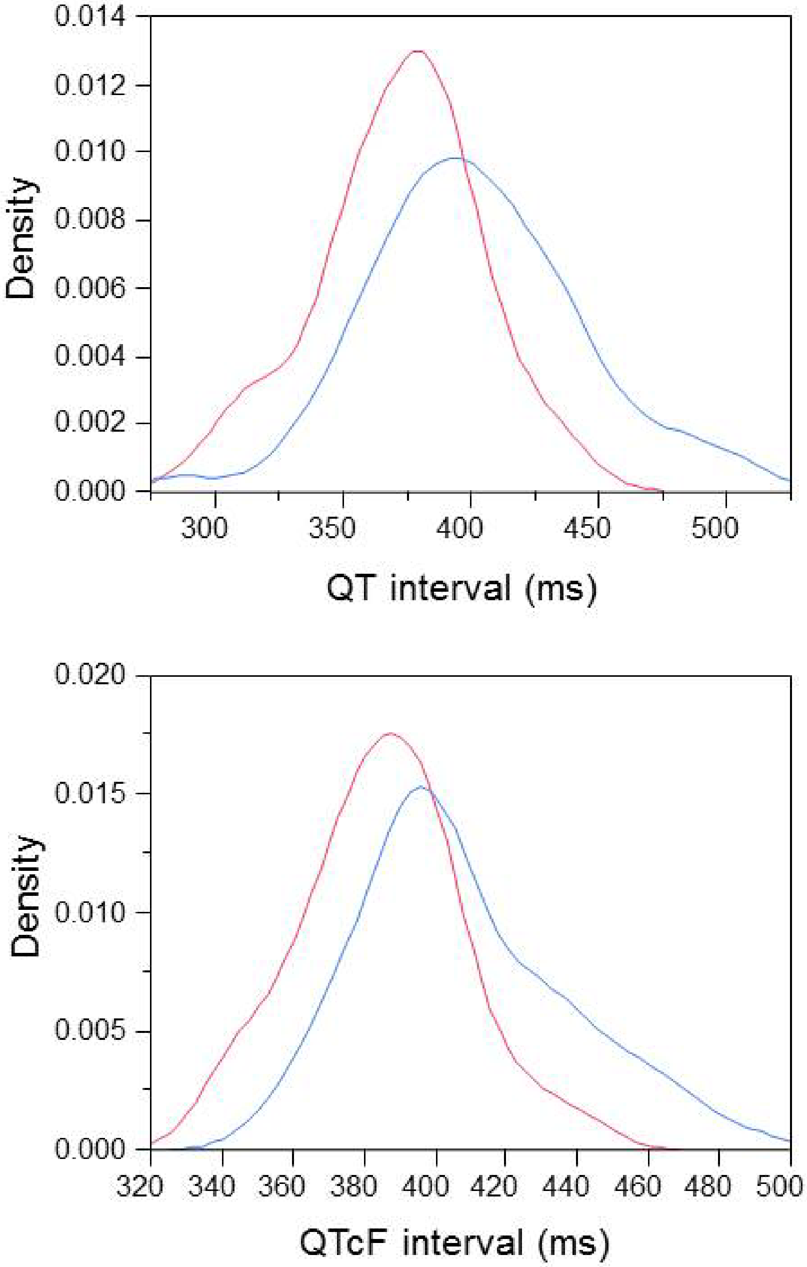

Supplemental Figure S2. QT (upper panel) and QTcF (lower panel) interval duration distribution in *TGFβR2* mutation carriers (blue) and their non-carrier relatives (red).
